# Can AI applied on MRI reliably predict shunt response in INPH? A comprehensive exploration of deep learning and radiomics approaches using preoperative MRI

**DOI:** 10.1371/journal.pone.0350335

**Published:** 2026-06-08

**Authors:** Klara Mogensen, Valerio Guarrasi, Sofia Behndig, Johan Eriksson De Ryst, Paolo Soda, Anders Eklund, Jan Malm, Sara Qvarlander

**Affiliations:** 1 Department of Diagnostics and Intervention, Biomedical Engineering and Radiation Physics, Umeå University, Umeå, Sweden; 2 Unit of Artificial Intelligence and Computer Systems, Department of Engineering, Università Campus Bio-Medico di Roma, Rome, Italy; 3 Department of Diagnostics and Intervention, Diagnostic Radiology, Umeå University, Umeå, Sweden; 4 Department of Clinical Sciences, Neurosciences, Umeå University, Umeå, Sweden; 5 Umeå Center for Functional Brain Imaging, Umeå University, Umeå, Sweden; University of Rochester, UNITED STATES OF AMERICA

## Abstract

**Objective:**

Idiopathic normal pressure hydrocephalus (INPH) is a treatable neurological condition, yet predicting which patients will benefit from a cerebrospinal fluid shunt remains challenging. Structural brain MRI is a core part of the diagnostic workup, but traditional radiological measures show limited predictive accuracy. This study aimed to assess whether deep learning and radiomics-based machine learning approaches can provide clinically useful predictions of shunt outcome based on preoperative MRI.

**Methods:**

We investigated 149 shunted INPH patients with available preoperative T1-weighted, T2-weighted, and FLAIR images. Patients were classified as responders (n = 113) or non-responders (n = 36) based on postoperative gait speed improvement. For INPH, this is a large sample with typical outcome distribution. Three artificial intelligence approaches were tested: a late-fusion ensemble of multiple 3D convolutional neural networks; a multimodal intermediate fusion model; and radiomics-based machine learning models trained on features extracted from whole-brain masks. Models were assessed using 10-fold cross-validation. The best performing model on the validation set was selected from each approach. Performance metrics included the area under the receiving operating characteristic curve (AUROC), sensitivity, and specificity.

**Results:**

Performance was considered poor in all models, and none reached an area under the receiving operating characteristic curve above 70%. Of the three methodologies, the best performance was achieved with a radiomics-based model (Linear Discriminant Analysis classifier on T1-weighted images) which achieved an AUROC of 63.7%. In a reduced subset of clearly separated responders and non-responders (n = 72), the best model (late fusion ensemble of 5 convolutional neural networks) reached an AUROC of 69.2%.

**Conclusions:**

Despite the use of advanced artificial intelligence techniques, structural MRI alone were insufficient for reliably predicting gait outcome after surgery in idiopathic normal pressure hydrocephalus. To capture the complexity of the condition and enable clinically meaningful predictions, our findings indicate the need for research investigating multimodal input and using large multi-center datasets.

## Introduction

Artificial intelligence (AI) and deep learning (DL) are transforming the way we use medical images, offering new opportunities for more reliable, objective, and scalable tools to aid in clinical decision-making. This type of approach shows promise in evaluating neurological conditions such as Alzheimer’s disease [1]. Another neurological syndrome, affecting older people, is idiopathic normal pressure hydrocephalus (INPH), which radiologically is characterized by enlarged cerebral ventricles [2]. This condition affects both men and women and becomes more common with age, with an estimated prevalence of 7% among the people over 80 years [3,4]. Patients present with a characteristic gait/balance disorder, often accompanied by cognitive decline and incontinence [5]. Particularly the gait can be improved with neurosurgical insertion of a cerebrospinal fluid (CSF) shunt [6]. INPH is thus a treatable disease, but clinical signs and symptoms overlap with several differential diagnoses, such as Parkinson’s and Alzheimer’s disease, that do not respond to shunting, highlighting a key challenge in INPH care: identifying patients that will improve after shunt surgery [7].

Brain imaging is an essential component of the diagnostic workup for INPH. Ventricular size and morphology measurements have been utilized not only for diagnosis but also to predict surgical outcome [5]. However, the scientific evidence supporting their predictive value remains conflicting [8]. Many approaches exist to improve the selection of INPH patients to surgery and invasive test are today common, in addition to radiology. In a meta-analysis [9], the authors recommend tap-test or infusion testing as first line of invasive test to predict shunt outcome, with an area under the receiving operating curve (AUROC) of 0.71 and 0.73 respectively. Regarding the radiological measures, another meta-analysis found that the callosal angle is the most reliable radiological predictor of shunt response (diagnostic odds ratio = 1.88), though its predictive value is still much lower than invasive tests (tap-test: DOR = 3.86 and infusion testing: DOR = 5.7) and the authors suggest that combining multiple radiological markers using machine learning (ML) may significantly enhance predictive accuracy [10]. This motivates investigation of whether advanced methods that make full use of the information available in the MR images can improve prediction of shunt responsiveness.

Two recently published studies support the application of AI to brain MRI in INPH. Using a 3D convolutional neural network (CNN) ensemble approach, it was demonstrated that AI could identify individuals in the general population who exhibit the characteristic gait pattern associated with INPH [11]. Another group applied 3D CNNs to dual-sequence brain MRI with somewhat promising results in predicting which patients are likely to benefit from treatment [12]. However, further advancements in performance are needed before such models can be clinically useful. Additionally, a recognized challenge in applying AI in radiology is the limited reproducibility of seemingly successful single-center studies [13], therefore confirmatory studies from other centers are needed.

In this study, we adopted a comprehensive approach to determine the effectiveness of AI on structural MRI to predict shunt outcome. We made use of the ensemble search method [11] applied to three standard brain MRI sequences, as well as an alternative DL methodology with intermediate fusion, which enables the model to learn joint patterns in the different sequences. We also tested several ML models, using radiomic features extracted from the images. Our aim was to explore whether suggested AI-based approaches could provide clinically useful predictions of shunt responsiveness in INPH patients.

## Method

In summary, we aimed to predict shunt outcome based on gait speed, in patients with INPH using preoperative brain MRI. We analyzed the images with three different methodologies (ensemble search with late fusion, intermediate fusion, and ML on radiomic data) and evaluated them using 10-fold cross validation for balanced accuracy (accounted for class imbalance), sensitivity, specificity, and AUROC.

Based on the idea that different networks might capture different aspects of the data [14] we performed a DL model ensemble search, based on 19 separately trained 3D CNN architectures. While the ensemble strategy applies late fusion at the decision level [11], previous work [12,15] suggests that intermediate fusion of different MRI sequences may have advantages for this type of problem. To explore this, we implemented an intermediate fusion method based on three (one per sequence) ResNet18 networks, connected by information-sharing modules. Considering the limited dataset and complexity of whole brain imaging, for comparison, we also implemented a traditional ML pipeline on radiomic features. Several commonly used ML classifiers were evaluated, after feature selection.

### Patient cohort

The cohort consisted of patients diagnosed with INPH during 2007–2019 at Umeå University Hospital. Those who underwent shunt surgery and had preoperative MRI with T1-weighted (T1w), T2-weighted (T2w), and FLAIR sequences available were included in the cohort. All patients were evaluated using a standardized clinical protocol, including neurological, gait, and cognitive assessments, and CSF pressure measurement to obtain the diagnosis. For shunt selection CSF infusion investigations and tap tests were also performed. A CELDA^®^ apparatus (Likvor AB, Umeå, Sweden) was used for lumbar CSF infusion testing to assess CSF dynamics, in particular the CSF outflow resistance. This was followed by a tap test where 30–50 ml of CSF was removed. If the surgical indication was uncertain after this evaluation, the patients were additionally assessed with external lumbar drainage. Surgery was offered to patients with a clinical and radiological presentation consistent with INPH [5], no alternative explanation for symptoms, and positive tap test and/or elevated CSF outflow resistance or positive response to external lumbar drainage. If patients presented with comorbidities, they were considered in the decision of shunt surgery and patients were offered a shunt if INPH was considered to be the main contributor to the symptoms.

In total, there were 259 shunted INPH patients during the time period who had post operative gait assessments available. Among these, 9 patients experienced adverse events (such as hip fractures or subdural hematomas), and 8 patients had a shunt malfunction (verified with an additional infusion test) which was discovered during the follow up assessments, excluding them from the study. As INPH is a progressive condition, the 11 patients who had more than 1 year between pre-operative assessment and surgery, or more than 2 years between surgery and the post-operative assessment were also excluded. Mean time from assessment to surgery was 139 days (SD = 81, range 1–363 days) and mean follow-up time 215 days (SD = 133, range 75–719 days).

Shunt response was determined based on maximum gait speed, assessed as average of up to six repetitions of a minimal distance of ten meters. Patients with at least 0.16 m/s increased gait speed postoperatively [16] were considered shunt responders, while those with less than 0.1 m/s increase were considered non-responders. Patients with increases of 0.1–0.16 m/s, as well as patients with <0.1 m/s improvement in gait speed but substantial improvements in balance were excluded (n = 30) to provide clear cut groups for model training. The cutoff of 0.16 m/s is based on a previous study [16] which estimated this to be the minimal clinically important difference for comfortable gait speed.

Among the remaining patients, 52 patients lacked at least one MRI sequence, which excluded them from the study.

In total, there were 149 included patients, of which 113 were considered shunt responders and 36 non-responders. For INPH, this is a large sample size with an expected outcome distribution [7]. Mean age of the responders and non-responders was 74.3 years (SD = 5.56) and 74.1 years (SD = 5.91), respectively (p = 0.900, two-sample t-test). Among responders 33.6% were female (n = 38) and for non-responders 27.8% (n = 10), (p = 0.644, Chi-square test).

The study was approved by the Swedish ethical review authority (no. 2020–04469) and conducted in accordance with the declaration of Helsinki. All patients were informed about the study by letter and had the possibility to opt out. Deceased individuals were included. Medical records were accessed for this study from 10-11-2020. During the data acquisition the authors had access to information that could identify individual participants.

### MR Images

Three brain MRI sequences T1w, T2w and FLAIR, were collected from the preoperative scans. As the study is retrospective, the images differed in size and resolution. Images with too low resolution or number of slices were excluded. Table 1 presents the averages along with the least favorable value for size and resolution of included images.

**Table 1:** Summary of MRI characteristics.

Sequence	Number of slices		Acquisition pixel size(frequency and phase direction) [mm]				Reconstructed pixel size (isotropic) [mm]		Slice thickness [mm]		Slice spacing [mm]	
	Mean	Min	Mean		Max		Mean	Max	Mean	Max	Mean	Max
			Freq.	Phase	Freq.	Phase						
T1w	190	165	0.738	0.908	1.04	1.28	0.362	1.00	1.00	1.10	1.00	1.10
FLAIR	58.4	37	0.833	1.03	1.56	1.50	0.529	1.04	2.95	4.00	2.99	4.00
T2w	49.0	25	0.569	0.737	0.898	1.12	0.445	0.718	3.11	5.00	3.21	6.00

The mean is accompanied by the worst-case observed value for each measure.

### Preprocessing

**Fig 1:**
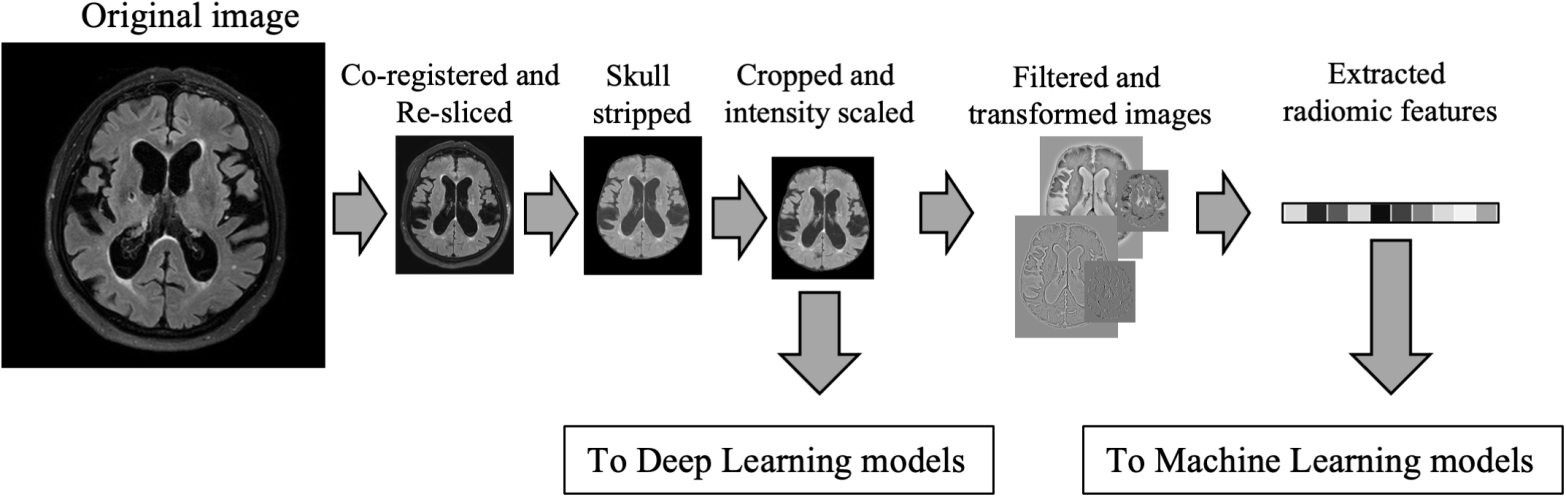
Preprocessing pipeline for both the Deep Learning (DL) and Machine Learning (ML) workflows. The process is here illustrated for one FLAIR image but the same steps were applied to all MRI sequences. After co-registration and re-slicing, all images were standardized to the same size. Skull stripping was performed to keep only the intracranial volume, followed by intensity scaling to the range [−1, 1], with background voxels set to −1. The images were additionally cropped to exclude redundant background. For ML models, Laplacian of Gaussian filters and wavelet transforms were applied to generate multiple image representations, from which radiomic features were extracted.

Fig 1 illustrates the preprocessing pipeline. All images underwent co-registration and re-slicing to the ICBM-152 template brain and bias field correction using SPM12 [17]. Images were skull stripped to retain only the intracranial volume using SynthStrip [18]. To mitigate scanner-related intensity differences, voxel intensities greater than 3 standard deviations above the mean were clipped, and all voxel intensities were scaled to range between −1 and 1. Background voxels were set to −1. After preprocessing, all images had uniform resolution of 1 mm isotropic. When fed to the DL networks, the size of each input image was 160x192x160 pixels.

For extracting radiomic features, preprocessed images were used with the brain treated as a single region of interest. Features were extracted separately for each sequence from original, wavelet-transformed, and Laplacian of Gaussian (LoG) filtered images, capturing intensity and texture characteristics across spatial resolutions.

### Neural networks

#### Ensemble search.

We trained 19 well-known 3D CNN architectures, including ResNet [19], DenseNet [20], SENet [21], and SFCN [22] independently on each MRI sequence, to predict shunt response. See S1 Table for the full list of networks, including pretraining. Among all 57 CNNs, those obtaining specificity and sensitivity above 60% on the validation set were included in the late fusion ensemble where their predictions were combined at decision level. A backward search algorithm [11], iteratively removed models based on ensemble performance (balanced accuracy, F1-score) and the diversity of their classification outputs (rho, kappa, and Q-statistic), identifying the most effective subset. This strategy enabled integration of modality-specific features without explicit feature sharing during training.

#### Multimodal model.

**Fig 2:**
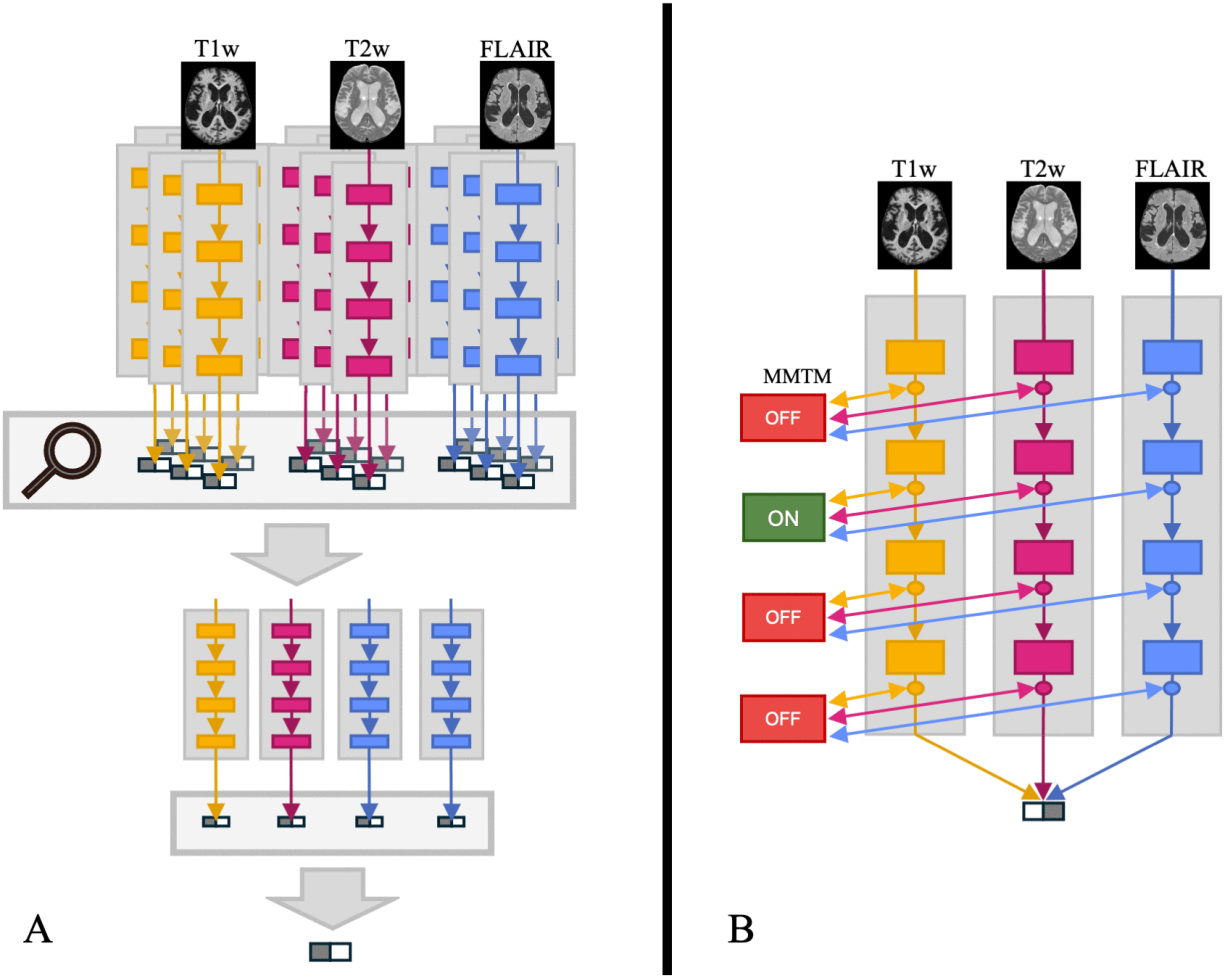
The two multisequence approaches. (A) For the ensemble search the process begins with multiple 3D convolutional neural networks, each independently trained on a single MRI sequence. Networks are removed one by one by a backwards search algorithm based on their contribution to overall performance and output diversity. The procedure stops when further removal no longer improves ensemble performance. Final predictions are aggregated at the decision level from the remaining networks.(B) The multimodal model is composed of three parallel ResNet-18 networks, each receiving one MRI modality (T1w, T2w, FLAIR) as input. The networks are interconnected by four Multimodal Transfer Modules (MMTMs), which enable information exchange between modalities. In the illustrated example, only the second MMTM is activated; at this point, features from all three streams are fused and redistributed to each network. The final classification is produced jointly from the outputs of all three streams. In total, six training configurations were evaluated: one for each MMTM activated individually, one with no MMTMs activated (i.e., independent streams), and one with all MMTMs activated.

We also trained a multimodal model which used an intermediate fusion strategy [15]. In brief, it consisted of three parallel 3D ResNet-18 streams, one per MRI sequence, interconnected by four Multimodal Transfer Modules [23] (MMTMs). These MMTMs can be selectively activated or deactivated; when all are deactivated, the model effectively behaves as three independent networks with a joint classification layer at the output. If an MMTM is active, information from all modalities is fused and fed back into each stream, allowing the model to learn from all sequences simultaneously. The model was tried with 6 different configurations: no MMTM active, each one of the four MMTMs active at a time, and all MMTMs active. Both the ensemble search and the multimodal model are illustrated in Fig 2.

#### Machine learning models.

The extracted radiomic features included first-order statistics, shape descriptors, and texture metrics derived from gray-level co-occurrence, run-length, size zone, tone difference, and dependence matrices [24]. All features were computed in 3D, using the full brain mask. We evaluated 25 commonly used ML classifiers, including Random Forest, Extreme Gradient Boosting, support vector machines, linear discriminant analysis and neural networks. See S2 Table for the full list.

### Technical setup

During all tests, class labels were binary (0 = non-responder, 1 = responder), and performance metrics were computed for each model to identify the best-performing approaches. All models were trained using a 10-fold stratified cross-validation scheme, using 8 folds for training, 1 for validation, and 1 for testing. To address class imbalance, we duplicated the non-responder cases in the training and validation set during training.

The results are presented in terms of AUROC to facilitate comparison with other studies, as it is commonly reported in clinical research. However, AUROC does not account for class imbalance. Overall accuracy is often considered the most intuitive performance metric, but because of the class imbalance, balanced accuracy was used instead, defined as the mean of specificity and sensitivity. To further illustrate the strengths and limitations of the different models, specificity and sensitivity are also presented to provide class specific performance metrics.

For the DL models, class weights were also applied in the loss function and we applied random data augmentation, including shifts (±3 voxels in all directions) and left–right flipping. Networks were trained with default hyperparameters, optimized using cross-entropy loss and Adam optimizer with an initial learning rate of 1 × 10 ⁻ ⁵, reduced by a factor of 10 if validation loss plateaued for 10 epochs. Training was limited to 300 epochs with early stopping after 50 epochs of no validation improvement.

The radiomic features were reduced via a selection process within each cross-validation fold. Low-variance features were removed and highly correlated features (Pearson’s |r| > 0.9) were filtered to minimize redundancy. Finally, a Random Forest classifier was used to rank feature importance, and the top 20 features were retained for model training.

DL models were implemented in Python, using PyTorch. Radiomic feature extraction was performed using PyRadiomics and ML models were implemented using scikit-learn and XGBoost. DL models were trained on four NVIDIA A40 GPUs (48 GB each), while ML models were trained locally.

## Results

### Performance on the full dataset

**Fig 3:**
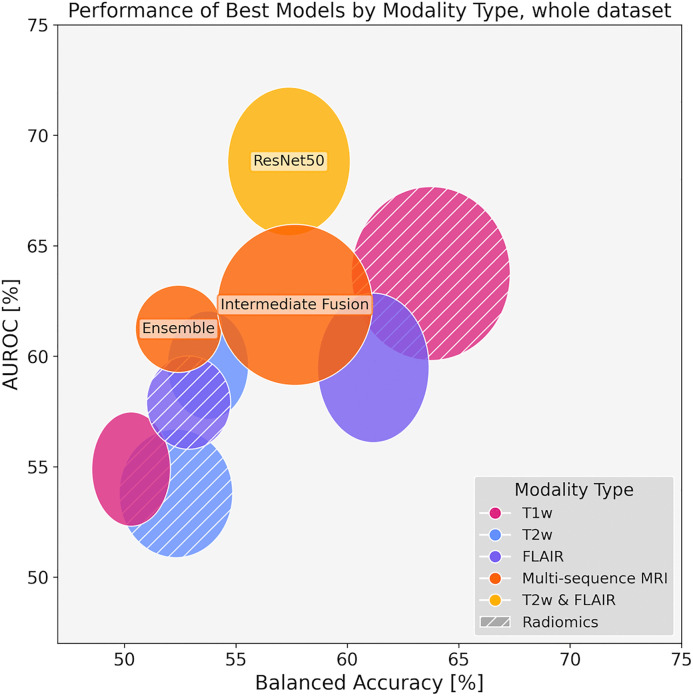
Test set performance on whole dataset. Mean balanced accuracy and AUROC on the test set over 10 folds of the full dataset (n = 149). The models shown are the best performers from each method and MRI sequence. The yellow ellipse corresponds to the results obtained by the ensemble consisting of ResNet50 for T2w and FLAIR, which was created specifically for comparison to a previous study [12]. The lengths of the axes correspond to the standard error of the mean for the balanced accuracy and AUROC respectively.

We selected the best model from each approach and modality based on the highest validation AUROC, results for the test sets are presented in Fig 3. The backwards search algorithm, resulted in an ensemble consisting of 4 CNNs; 1 using T1w, 2 using T2w and 1 using FLAIR (see Table 2). For the multimodal model with intermediate fusion, the best result was achieved with one MMTM activated on the second position. Detailed results are presented in Table 2. Among all models, none reached an AUROC above 70%, which can be considered poor performance [25]. The best-performing model in terms of both balanced accuracy and AUROC was the Linear Discriminant Analysis classifier based on radiomic features from T1w with 63.74% and 63.75% respectively. As a comparison, we also tested the ensemble consisting of ResNet50 for T2w and FLAIR, resembling the approach of a previous study [12], which had a marginally better AUROC, but performed worse in other metrics.

**Table 2:** Test set performance for selected models on the full dataset (shunt responder = 113, non-responder = 36).

Sequence	Type/Feature	Model	AUROC	Balanced accuracy	Sensitivity	Specificity
Multi-sequence MRI	3D MRI	Intermediate fusion*	62.32 ± 23.07	57.65 ± 22.02	63.64 ± 17.68	51.67 ± 34.65
Multi-sequence MRI	3D MRI	Ensemble**	56.72 ± 16.33	54.89 ± 14.26	72.27 ± 20.49	37.5 ± 40.3
FLAIR	3D MRI	DenseNet264	59.47 ± 21.4	61.17 ± 15.84	61.52 ± 32.83	**60.83 ± 29.93**
FLAIR	Radiomic features	XGBoost Random Forest	57.9 ± 13.37	52.88 ± 11.94	79.92 ± 10.95	25.83 ± 22.72
T1w	3D MRI	SEResNet50	54.89 ± 16.36	50.3 ± 11.18	70.61 ± 29.9	30 ± 34.29
T1w	Radiomic features	Linear Discriminant Analysis	**63.74 ± 24.9**	**63.75 ± 22.57**	**80.00 ± 12.11**	47.5 ± 38.9
T2w	3D MRI	ResNet34	59.59 ± 15.55	53.75 ± 11.53	75.84 ± 24.92	31.67 ± 28.81
T2w	Radiomic features	K-Nearest Neighbors	53.79 ± 18.41	52.31 ± 16.13	77.96 ± 16.21	26.67 ± 30.88
T2w & FLAIR	3D MRI	Late fusion with ResNet50	**68.81 ± 21.29**	57.39 ± 17.4	61.44 ± 30.56	53.33 ± 36.47

The results are presented as mean and standard deviation over the 10 folds. The best performing model for each metric is highlighted. The shaded model was added specifically for comparison to a previous study [8].

* Second multimodal transfer module active.

** Included networks: SEResNet50 (T1w), SEResNeXt101 (FLAIR), ResNet101 (T2w), ResNet50 (T2w).

Among the radiomic features, those derived from 3 mm LoG filtered images and gray level run length matrix (GLRLM) metrics were most frequently incorporated into the ML models for T2w and FLAIR. Specifically, the LoG-based Short Run Low Gray Level Emphasis was selected in all 10 folds for FLAIR, while Long Run Low Gray Level Emphasis was consistently selected in all 10 folds for T2w images. The LoG-based gray level co-occurrence matrix (GLCM) Cluster Prominence feature was also present across all folds for T2w. For T1w, the most frequently selected features were wavelet-derived, with GLCM Difference Entropy being selected in 9 out of 10 folds. The 20 selected features varied across folds, in total 92 radiomic features were selected in at least on fold for T1w, 94 for T2w and 102 for FLAIR.

### Most improved responders for balanced groups

**Fig 4:**
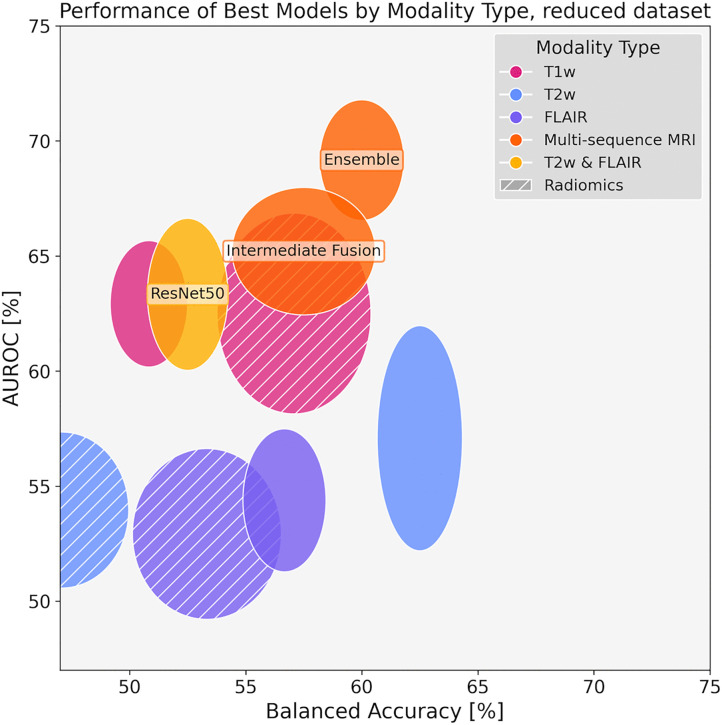
Test set performance on the reduced dataset. The figure shows mean balanced accuracy and AUROC on the test set over 10 folds, using the most extreme shunt responders and all non-responders (n = 72). The models shown are the best performing from each method and MRI sequence. The yellow ellipse corresponds to the results obtained by the ensemble consisting of ResNet50 for T2w and FLAIR, which was created specifically for comparison to a previous study [12]. The lengths of the axes correspond to the standard error of the mean for the balanced accuracy and AUROC respectively.

If brain morphology is related to symptom severity, or shunt outcome, making the group separation clearer would potentially facilitate the models’ ability to identify the important features. To explore whether clearer group separation would improve model performance, we conducted a post-hoc test where we repeated the experiments on a reduced subset of the data. This subset included all 36 non-responders and the 36 shunt responders with the highest improvement in gait speed (Δ > 0.42 m/s). Fig 4 presents mean balanced accuracy and AUROC scores across folds for the best models from each modality.

Table 3 summarizes the detailed results for the subset. Overall performance remained modest in all models. The best-performing model in terms of balanced accuracy was ResNet34 on T2w, achieving 62.50%, and in terms of AUROC, the ensemble model based on five CNNs, at 69.17%. Thus, despite better separation of the groups in the training data, the models did not achieve clinically reliable predictive performance.

**Table 3:** Test set performance on reduced dataset (shunt responder = 36, non-responder = 36).

Sequence	Type/Feature	Model	AUROC	Balanced accuracy	Sensitivity	Specificity
Multi-sequence MRI	3D MRI	Intermediate fusion*	65.21 ± 17.51	57.5 ± 19.52	**66.67 ± 21.87**	48.33 ± 29.87
Multi-sequence MRI	3D MRI	Ensemble**	**69.17 ± 16.57**	60 ± 11.49	59.17 ± 27.06	**60.83 ± 24.86**
FLAIR	3D MRI	ResNet10	54.38 ± 19.65	56.67 ± 11.32	55.83 ± 33.11	57.50 ± 41.66
FLAIR	Radiomic features	Gaussian Naïve Bayes	52.92 ± 23.47	53.33 ± 20.3	48.33 ± 30.38	58.33 ± 24.53
T1w	3D MRI	DenseNet201	62.92 ± 17.37	50.83 ± 10.54	62.5 ± 30.99	39.17 ± 32.88
T1w	Radiomic features	XGBoost Random Forest	62.50 ± 27.59	57.08 ± 20.98	59.17 ± 24.99	55.00 ± 29.45
T2w	3D MRI	ResNet34	57.08 ± 30.94	**62.50 ± 11.62**	75.00 ± 27.5	50.00 ± 34.25
T2w	Radiomic features	Logistic Regression	53.96 ± 21.44	47.08 ± 18.22	43.33 ± 31.87	50.83 ± 26.77
T2w & FLAIR	3D MRI	Late fusion with ResNet50	63.33 ± 20.88	52.50 ± 10.97	64.17 ± 18.86	40.83 ± 32.26

The results are presented as mean and standard deviation over the 10 folds. The best performing network for each metric is highlighted.

*All multimodal transfer modules active.

**Included networks: ResNet101 (T1w), DenseNet121 (T1w), ResNet101 (T2w), ResNet152 (T2w), DenseNet264(T2w).

In the reduced dataset, wavelet-derived features dominated among the selected radiomic features. For T1w, the GLCM Difference Entropy was again the most frequently selected feature, appearing in 9 of 10 folds. For FLAIR images, wavelet-based first-order Skewness was selected in 8 of 10 folds, while for T2w, GLRLM High Gray Level Run Emphasis was selected in 7 of 10 folds. The number of total features used in any fold was similar to when the whole dataset was used with 92 for T1w, 106 for T2w and 103 for FLAIR.

## Discussion

In this study, we investigated a range of DL and radiomics-based ML models to predict shunt response, in terms of gait speed improvement, in patients with INPH using preoperative MRI. Despite exploring multiple architectures and modalities, no model achieved clinically useful predictive performance. This suggests that the predictive information in the structural MRI is limited, making it difficult to capture the complexity of the condition. Our findings thus indicate that supplementary data or substantially larger datasets are needed to create reliable prediction models that can improve current clinical practice for INPH.

The proposed ensemble search model, which had previously shown good results on a similar task [11], did not manage to predict shunt outcome and neither did the radiomics-based models. The best result on the whole dataset, in terms of AUROC and balanced accuracy, was obtained by a linear discriminant analysis classifier on radiomic features from T1w images, with 64%. Among all models, the highest AUROC was obtained by the ensemble model applied to the reduced dataset, achieving 69%. That the reduced dataset did not yield better results implies that a critical factor may be the size of the non-responder group, rather than group separation or class imbalance. This is further supported by the observation that different radiomic features were selected in the reduced dataset. This may indicate that the data heterogeneity did not decrease in the reduced group of improvers, rather that the smaller fold sizes may have limited the model’s ability to generalize. This may be promising for future research since class imbalance is natural for INPH cohorts, given that selecting the appropriate patients for surgery is part of the clinical routine. Increasing the group size on the other hand can be managed by multi-center studies. Addressing such factors is needed, since the present results are not good enough to substantially improve the current established clinical practice for shunt investigation [7,9,10].

The ensemble search model has previously, in a population-based study, shown good results in identifying INPH-typical gait impairment compared to controls, based on brain MRI [11], but on this much more challenging task it did not perform as well. Additionally, Leary et al. reported encouraging results (AUROC = 88%) using 3D CNNs on dual-sequence MRI for predicting shunt response [12]. However, our attempt to replicate this approach using a wider set of models and a larger modality combination (including T1w) did not yield comparable results. Nor did using only the same sequences and network [12]; the ResNet50 ensemble obtained slightly better results in AUROC, but performed comparably in other metrics. Our results could be seen as surprising, given the successful outcome in these previous studies, but there are some possible explanations. First, it is important to distinguish diagnostic and predictive tests. Classifying our study participants compared to controls is likely an easier task, since radiology is an essential part of the diagnostic workup in INPH [5,26], whereas the role of structural MRI is more debated in prediction [10]. Second, in our cohort, in contrast to the Leary cohort, the patients have undergone a more extensive clinical protocol including tap test and infusion testing before being selected for shunt surgery. This means we are investigating what more information there are in the images, that can contribute to outcome prediction, beyond the information from these tests. This is probably a more complicated task than investigating which information the images can contribute with, with no additional invasive tests.

Other differences than cohort composition between our study and the one by Leary et al., are outcome definitions and model training protocols. While the previous study used the categorical scoring of the INPH scale [27] for assessing the gait impairment, the present study used maximum gait speed improvement as the outcome measure. Data collection began before the INPH scale was published, consequently it is not applicable for this cohort, preventing a direct comparison with the same outcome metric. Gait and balance are the only symptoms in the INPH triad that are proven to improve by shunting in a randomized, placebo-controlled study, with improvement in gait speed showing the strongest effect [6]. This is therefore a well suited outcome measure for testing these types of alternative approaches to determine shunt outcome. Furthermore, given the extended inclusion period of our study, use of a prospectively and objectively assessed quantitative metric is a strength.

Regardless of the specific reason behind the discrepancy in results, this highlights the challenges of reproducibility and generalizability in AI-based medical imaging research.

Our results could also be explained simply by the fact that although we used a relatively large cohort in the context of INPH research, the sample size is limited from a DL perspective. This may have constrained the models’ ability to generalize, especially when trying to learn high-dimensional features from volumetric MRI data. On the other hand, current radiological measures also have limited predictive value [10], which suggests that structural MRI alone may be insufficient for reliable prediction of shunt outcome in INPH. Shunt response is complex and can be influenced by many factors [7] that are not visible in preoperative structural MRI. For example, comorbidities, CSF dynamics [28], or subtle cognitive and gait features may contribute significantly to patient outcomes, yet potentially remain undetectable on conventional imaging. To determine the reason, bigger datasets are needed in future studies.

From a methodological perspective, this study has several strengths. We used consistent preprocessing and systematically compared a broad array of models, including 19 DL architectures and 25 ML methods, across multiple MRI sequences. We also explored both late and intermediate fusion strategies for multimodal modeling and investigated model performance on a reduced, more clearly defined patient subset. All the models performed quite poorly, supporting our negative conclusion.

There are some limitations worth noting. The analysis was restricted to structural MRI and did not incorporate clinical data, CSF biomarkers, or other potentially informative features [7,26]. Additionally, although follow-up assessments were standardized, shunt response was defined using a single gait speed metric, with an absolute cut-off for improvement (0.16 m/s). For reference, we note that if the improvement was expressed in percentage or preoperative gait speed (relative improvement), this corresponded to the same group division with a cutoff of 12%. Other dimensions of improvement, such as cognitive or urinary symptoms, were not included in the outcome definition, with the exception of excluding those patients who substantially improved in balance but not in gait speed. Typically, improvements in cognition and urinary symptoms are less common than improvement in gait following shunt surgery [29–31], and gait and balance are the only symptoms proven to improve randomized, placebo-controlled study [6], which supports our focus on this symptom. Furthermore, while external validation is generally desirable to assess generalizability, the limited performance observed in the main cohort indicates that no strong predictive pattern was identified. In this context, external validation would primarily have confirmed the modest performance rather than altering the overall interpretation. Lastly, the number of shunt responders was substantially larger than the number of non-responders, resulting in an imbalanced dataset. While this reflects the general situation for existing INPH cohorts rather than a limitation unique to our study, such imbalance can impact model training and evaluation. To mitigate this, we oversampled the non-responder group during training and used class-weighted loss functions.

Future research, if focusing solely on imaging, will require substantially larger datasets. To give predictive models a fair opportunity to learn meaningful patterns, data must capture the full spectrum of imaging presentations in INPH. The extracted radiomic features on the whole dataset were mainly describing texture patterns, characteristics that are difficult to quantify manually, which motivates the use of DL also in future models. However, considering the incompletely understood pathophysiology of INPH, imaging alone is unlikely to yield clinically useful predictive models for shunt outcome and a multimodal approach will probably be necessary. Integrating clinical and physiological data is likely essential. In addition to age, and duration and severity of symptoms, relevant variables could also include CSF outflow resistance, quantitative measures of improvement following the tap test or biomarkers from CSF [32]. Regarding the imaging, incorporating segmented volumes or quantitative measures of the shapes of the brain and the CSF spaces could also be of value. Using these additional features as input to a network, together with brain MRI, would more closely mirror the clinical work-up for INPH patients today. Although none of these measures alone can reliably predict shunt outcome [9,10], a combined model might identify patterns of how these features relate, potentially creating a stronger predictive model. Such multimodal approaches are therefore likely better positioned to capture the complexity of INPH and may offer more robust and clinically useful predictions. Bigger datasets would still be needed to capture the heterogeneity of the patient group, in particular the non-responders. Thus achieving this will likely require multi-center collaboration with harmonized clinical protocols and outcome measures to ensure consistency and generalizability.

## Conclusion

In conclusion, our findings suggest that image-based AI models are currently not reliable enough for predicting gait outcome after shunt surgery for INPH patients in a clinical setting. Given that the current radiological measures also have limited predictive value, these findings suggest that AI on structural MRI provides limited additional predictive value for shunt outcome in INPH patients pre-selected using tap test and infusion testing. To capture the complexity of the condition and enable clinically meaningful predictions, our findings indicate the need for research implementing multimodal input and large multi-center datasets.

## Supporting information

S1 Table Summary of tested 3D convolutional neural networks used in the backwards search and their pretraining. (DOCX)

S2 Table Summary of traditional machine learning classifiers evaluated in this study. The second column states their corresponding implementation names in the scikit-learn Python library. All classifiers were trained from scratch without pretraining. Note: XGBClassifier and XGBRFClassifier are implemented in the XGBoost library but follow the scikit-learn API.
(DOCX)
