# Supplementary material for: Can AI applied on MRI reliably predict shunt response in INPH? A comprehensive exploration of deep learning and radiomics approaches using preoperative MRI
Source: PLoS One. 2026 Jun 8;21(6):e0350335. doi: 10.1371/journal.pone.0350335 (PMC13245759; doi:10.1371/journal.pone.0350335)
Supplement: S1 Table — (DOCX) [file pone.0350335.s001.docx]

**S 1 Table. Summary of tested 3D convolutional neural networks used in the backwards search and their pretraining.**

| Comment | Name | Pretraining |
| --- | --- | --- |
| Residual Network | ResNet10 | Med3D^[[1]](#footnote-1)^ (23 medical datasets). |
|  | ResNet18 | Med3D |
|  | ResNet34 | Med3D |
|  | ResNet50 | Med3D |
|  | ResNet101 | Med3D |
|  | ResNet152 | Med3D |
|  | ResNet200 | Med3D |
| Squeeze and Excitation Network | SENet154 | ImageNet^[[2]](#footnote-2)^ |
|  | SEResNet50 | ImageNet |
|  | SEResNet101 | ImageNet |
|  | SEResNet152 | ImageNet |
|  | SEResNeXt50 | ImageNet |
|  | SEResNeXt101 | ImageNet |
| Dense Convolutional Network | DenseNet121 | None |
|  | DenseNet169 | None |
|  | DenseNet201 | None |
|  | DenseNet264 | None |
| Simple Fully Convolutional Network | SFCN | MRI for age prediction^[[3]](#footnote-3)^ (UK biobank^[[4]](#footnote-4)^). |
|  | SFCNFC  (FC = final convolutional head replaced by two fully connected layers) | MRI for age prediction. |

1. Chen S, Ma K, Zheng Y. Med3D: Transfer Learning for 3D Medical Image Analysis. Published online July 17, 2019. doi:10.48550/arXiv.1904.00625 [↑](#footnote-ref-1)
2. Deng J, Dong W, Socher R, et al. ImageNet: A large-scale hierarchical image database. In: 2009 IEEE Conference on Computer Vision and Pattern Recognition. IEEE; 2009:248-255. doi:10.1109/CVPR.2009.5206848 [↑](#footnote-ref-2)
3. Peng H, Gong W, Beckmann CF, et al. Accurate brain age prediction with lightweight deep neural networks. Medical Image Analysis. 2021;68:101871. doi:10.1016/j.media.2020.101871 [↑](#footnote-ref-3)
4. Miller KL, Alfaro-Almagro F, Bangerter NK, et al. Multimodal population brain imaging in the UK Biobank prospective epidemiological study. Nat Neurosci. 2016;19(11):1523-1536. doi:10.1038/nn.4393 [↑](#footnote-ref-4)
