# Supplementary material for: Can AI applied on MRI reliably predict shunt response in INPH? A comprehensive exploration of deep learning and radiomics approaches using preoperative MRI
Source: PLoS One. 2026 Jun 8;21(6):e0350335. doi: 10.1371/journal.pone.0350335 (PMC13245759; doi:10.1371/journal.pone.0350335)
Supplement: S2 Table — The second column states their corresponding implementation names in the scikit-learn Python library. All classifiers were trained from scratch without pretraining. Note: XGBClassifier and XGBRFClassifier are implemented in the XGBoost library but follow the scikit-learn API. (DOCX) [file pone.0350335.s002.docx]

**S 2 Table. Summary of traditional machine learning classifiers evaluated in this study.**

| Scientific name | scikit-learn class name |
| --- | --- |
| Adaptive Boosting Classifier | AdaBoostClassifier |
| Bernoulli Naive Bayes Classifier | BernoulliNB |
| Decision Tree Classifier | DecisionTreeClassifier |
| Extremely Randomized Tree Classifier | ExtraTreeClassifier |
| Ensemble of Extremely Randomized Trees Classifier | ExtraTreesClassifier |
| Gaussian Naive Bayes Classifier | GaussianNB |
| Gaussian Process Classifier | GaussianProcessClassifier |
| Gradient Boosting Classifier | GradientBoostingClassifier |
| Histogram-Based Gradient Boosting Classifier | HistGradientBoostingClassifier |
| k-Nearest Neighbors Classifier | KNeighborsClassifier |
| Linear Discriminant Analysis Classifier | LinearDiscriminantAnalysis |
| Nu-Support Vector Machine Classifier | LinearSVC |
| Logistic Regression Classifier | LogisticRegression |
| Multi-Layer Perceptron Classifier | MLPClassifier |
| Nearest Centroid Classifier | NearestCentroid |
| Nu-Support Vector Classifier | NuSVC |
| Passive-Aggressive Classifier | PassiveAggressiveClassifier |
| Perceptron Classifier | Perceptron |
| Quadratic Discriminant Analysis Classifier | QuadraticDiscriminantAnalysis |
| Random Forest Classifier | RandomForestClassifier |
| Ridge Classifier | RidgeClassifier |
| Stochastic Gradient Descent Classifier | SGDClassifier |
| Support Vector Machine Classifier (Kernel-based) | SVC |
| Extreme Gradient Boosting Classifier | XGBClassifier |
| Random Forest Classifier using Extreme Gradient Boosting Backend | XGBRFClassifier |

The second column states their corresponding implementation names in the scikit-learn Python library**.** Note: XGBClassifier and XGBRFClassifier are implemented in the XGBoost library. All classifiers were trained from scratch without pretraining.
